# Skin patch delivery of a SARS-CoV-2 spike DNA vaccine produces broad neutralising antibody responses

**DOI:** 10.1016/j.heliyon.2025.e42533

**Published:** 2025-02-07

**Authors:** Christopher L.D. McMillan, Andrea V. Corner, Danushka K. Wijesundara, Jovin J.Y. Choo, Daraporn Pittayakhajonwut, Indrajeet Poredi, Rhys H. Parry, Guneet K. Bindra, Kimberley L. Bruce, Alexander A. Khromykh, Germain J.P. Fernando, Laurent Dapremont, Paul R. Young, David A. Muller

**Affiliations:** a School of Chemistry and Molecular Biosciences, The University of Queensland, Brisbane, QLD, 4072, Australia; b Australian Infectious Diseases Research Centre, Global Virus Network Centre of Excellence, Brisbane, QLD, 4072, Australia; c Vaxxas Biomedical Facility, Brisbane, QLD, 4007, Australia; d BioNet-Asia, Hi-Tech Industrial Estate, 81 Moo 1, Baan-Lane, Bang Pa-In, Ayutthaya, 13160, Thailand; e Technovalia Pty. Ltd. Mulgrave, VIC, 3170, Australia

**Keywords:** High density microarray patch (HD-MAP), SARS-CoV-2, DNA, Vaccine

## Abstract

The ongoing SARS-CoV-2 pandemic continues to be a major health burden globally, especially in resource-limited areas. Continued research into more effective and accessible vaccines is required to reduce the burden of disease. Here, we use an emerging vaccine delivery system, the high-density microarray patch (HD-MAP) to deliver a plasmid DNA vaccine (Delta 6P) encoding for the SARS-CoV-2 spike protein. HD-MAP delivery of this vaccine resulted in robust IgG responses in mice against multiple domains of the spike protein. The cellular response to vaccination was also measured, and comparative analysis showed that relative to intramuscular vaccination, HD-MAP vaccination elicited spike-specific CD4^+^ T and CD8^+^ T cell responses that were largely comparable, but the number of polyfunctional CD4^+^ T cells was higher in the HD-MAP group. Collectively, this work suggests that HD-MAP delivery of the Delta 6P vaccine is effective against SARS-CoV-2, warranting further investigation.

## 1 Introduction

Since the emergence of severe acute respiratory syndrome coronavirus 2 (SARS-CoV-2), in December 2019, a recorded 775 million cases and an estimated 7.1 million deaths have occurred globally [1]. Whilst the pandemic has transitioned to endemicity, this does not alleviate the severity of disease burden, reduce resurgences of seasonal infections, or lessen the threat of novel immune escape variants [[2], [3], [4], [5], [6]]. To ameliorate both symptoms and transmissibility of emerging SARS-CoV-2 variants, continual prophylactic and therapeutic research and development is vital. During the pandemic, multiple vaccines were rapidly developed as various modalities, such as protein subunit; inactivated virus; viral vector; nanoparticle; DNA; and mRNA [[7], [8], [9], [10], [11], [12], [13], [14], [15], [16], [17], [18]]. Although differing vaccines yielded variable protective efficacy, the cumulative effect of herd immunity successfully minimised waves of SARS-CoV-2 infection. However, recent research indicates waning population immunity when measuring neutralisation from sera of vaccinated individuals [19,20]. These observations are attributed to the continual mutations of the spike glycoprotein, which result in enhanced infectivity and immune evasion. This is concerning as the spike glycoprotein is the primary target of vaccine-induced neutralising antibodies [3,[21], [22], [23]]. Furthermore, other challenges exist with current vaccines available on the market. Particularly, mRNA vaccines require ultra-low storage temperature to retain vaccine stability. These limitations hinder global transport and distribution, which must be addressed to accomplish vaccine equity in resource-limited nations [14].

To overcome programmatic and logistical barriers to mass vaccination campaigns, alternative vaccine delivery methods should be explored. One such vaccine delivery system is the high-density microarray patch (HD-MAP). The HD-MAP is a square one-by-one centimetre polymer patch containing 5000 micro-projections/cm^2^, each 250 μM length [24]. The vaccine is coated onto the tips of micro-projections and applied to the skin at 20 m/s using a spring-loaded applicator [24]. This application's velocity enables the micro-projections to penetrate the stratum corneum and deposit the vaccine into the (epi)dermal layers of skin, which are rich in immune cells. As a function of the dynamic application coupled with the co-localisation of vaccine with immune cells, HD-MAP vaccine delivery has routinely been shown to result in enhanced immune responses [[25], [26], [27]]. Beyond the enhanced vaccine immune responses observed, microarray patches alleviate many of the challenges associated with vaccine distribution [28]. These improvements include a more stable vaccine profile, fewer required doses, elimination of cold-chain transport requirements, and easier application [24,29,30]. The administration of HD-MAPs does not require specialised medical personnel, promoting accessibility, convenience, and the potential for self-administration [31,32].

mRNA vaccines have been effective against COVID-19, though their instability outside of cold-chain storage and the continuous evolution of the virus pose challenges [33,34]. As a potential alternative, we have investigated a stable DNA vaccine targeting the Delta variant. DNA vaccines have been shown to be immunogenic, eliciting both robust cellular and antibody responses whilst avoiding stimulation of vector-specific antibodies [7,13,17]. Other advantages of DNA plasmids include the adaptability of the genetic sequence, providing the potential to quickly protect against mutating viral variants; and the possibility of prime-boost and multiple booster vaccination regimes [11]. We conducted dose-match comparative studies and assessed immune cell responses from the Delta 6P DNA vaccine administered via HD-MAP and intramuscular (IM) injection in mice.

The Delta 6P plasmid encodes for the SARS-CoV-2 Delta HexaPro spike protein, consisting of six proline substitutions and a mutated furin cleavage site. Previous studies have found that the addition of six prolines greatly increases the stabilisation of the spike protein in its prefusion conformation and enhances expression efficiency compared to previous generations [35]. The notable effects of HexaPro as a vaccine immunogen include, but are not limited to, the induction of robust CD4^+^ T helper 1 (Th1) cells and significantly increased antibody responses [36]. Furthermore, studies have identified that the SARS-CoV-2 HexaPro spike glycoprotein provides complete protection against viral challenge after a single dose [37]. Additionally, delivery with the HD-MAP resulted in significantly improved immunological cellular responses when compared to intradermal needle and syringe [38].

We propose Delta 6P as a potential DNA vaccine for SARS-CoV-2 with HD-MAP delivery as an alternative for electroporation and liquid jet injectors [16,39,40]. The immunogenicity of Delta 6P delivered by the HD-MAP or IM was assessed in animal models. Whilst similar antibody levels were induced compared to the IM injection groups, we observed significantly higher antibody levels in the HD-MAP groups when evaluated against the RBD of the SARS-CoV-2 Delta variant. The vaccine-elicited T cell response was evaluated and compared between HD-MAP and IM cohorts *in vivo* relative to the activity of T helper (Th) cells and cytotoxic T cells, and *in vitro* using a conventional intracellular cytokine staining (ICS) analysis. Comparative analysis showed that IM vaccination elicited higher Delta spike-specific T helper cell response, but not cytotoxic T cells. The ICS analysis showed that spike-specific CD4^+^ T and CD8^+^ T cell responses were largely comparable, but the number of polyfunctional CD4^+^ T cells was higher in the HD-MAP group. Collectively, the data suggest that the spike-specific CD4^+^ and CD8^+^ T cell response was largely similar between HD-MAP and IM groups.

## 2 Methods

### 2.1 Animal ethics and animals

This study was performed according to the mandated regulations set by the National Health and Medical Research Council of Australia (Australian Code of Practise for the Care and Use of animals for Scientific Purposes, 8th edition 2013). All animal procedures and protocols were approved and conducted in accordance with the University of Queensland Animal Ethics Committees (AEC), AEC approval number: 2021/AE000340. All work with SARS-CoV-2 was performed under the University of Queensland Institutional Biosafety Committee (UQ IBC) approval number: IBC/501B/SCMB/2021.

Female BALB/c mice were purchased from Australian BioResources (Moss Vale, Australia) and housed in pathogen-free environment at the Australian Institute for Bioengineering and Nanotechnology (AIBN) within the University of Queensland. Female age-matched 6 weeks old BALB/c mice were acclimatised for 7 days before experimental use.

### 2.2 Plasmids

The plasmid encoding the SARS-CoV-2 Delta spike protein was designed by Technovalia, Mulgrave, Australia and produced by BioNet Asia, Bangkok, Thailand. Plasmids encoding other SARS-CoV-2 spike proteins used for protein production for analysis of antibody responses were generated as previously described [37]. The plasmid encoding SARS-CoV-2 S HexaPro was a gift from J. McLellan (Addgene plasmid no. 154754; https://www.addgene.org/154754/). The Beta and Delta HexaPro constructs contained the following mutations relative to the original SARS-CoV-2 HexaPro construct: Beta - L18F, D80A, D215G, ΔL242, ΔA243, ΔL244, R246I, K417N, E484K, N501Y, D614G, A701V, Delta - T19R, G142D, Δ156E, Δ157F, R158G, L452R, T478K, D614G, P681R, and D950N).

### 2.3 Cell lines

Expi293-F and ExpiCHO-S cells (Thermo Fisher Scientific, Waltham, Massachusetts, USA) were purchased and maintained as per manufacturer's recommendations. VeroE6 cells (ATCC, Gaithersburg, Maryland, USA) were maintained in a humidified incubator at 37 °C with 5 % CO_2_ in Dulbecco's modified eagle medium (DMEM, Gibco, Los Angeles, California, USA) supplemented with 10 % FCS (Bovogen, Keilor East, Victoria, Australia) and sodium pyruvate (Gibco, Los Angeles, California, USA).

### 2.4 Protein expression and purification

The regions encoding the receptor binding domain (RBD) and N-terminal domain (NTD) of the spike protein was fused to human Fc and plasmids purified using the PureYield™ Plasmid Midi-prep system (Promega, Madison, Wisconsin, USA). Plasmid DNA was sterilised using a 0.22 μM filter before transfection into ExpiCHO-S cells (Thermo Fisher Scientific, Waltham, Massachusetts, USA) as per manufacturer's protocols. Expression was performed in 25 or 200 mL volumes, with 1 μg of plasmid transfected per mL of culture. Cell culture supernatant was harvested day 7 post transfection and then purified via Protein A affinity chromatography on an AKTA Start FPLC system (Cytiva, Marlborough, Massachusetts, USA), as per the manufacturer's recommendations. Purified proteins were buffered exchanged into phosphate buffered saline (PBS) using a 30-kDa molecular weight Amicon® Ultra Centrifugal Filter (Merk, Rahway, New Jersey, USA) with centrifugation at 4000×*g* at 4 °C, before storage at a temperature of −20 °C.

The Wild-type, Beta, and Delta SARS-CoV-2 spike HexaPro plasmids were processed as described above and transfected into Expi293-F cells, in 25 mL or 200 mL cultures, following the manufacturer's procedures. Cell culture supernatant was harvested, and the protein purified using an in-house made 2M-10B11 monoclonal antibody conjugated Hi-Trap Affinity Column as described [38].

### 2.5 Recombinant antibody expression and purification

Antibody heavy and light chain plasmids were prepared using the PureYield™ Plasmid Midi-kit system (Promega, Madison, Wisconsin, USA) and sterilised using a 0.22 μM filter. Plasmids were then transfected in ExpiCHO-S cells as before, following the manufacturer's protocol. On day 7 post-transfection, antibodies were purified using a Protein A affinity column and buffer exchanged as described above.

### 2.6 Mouse immunisations

**Fig. 1:**
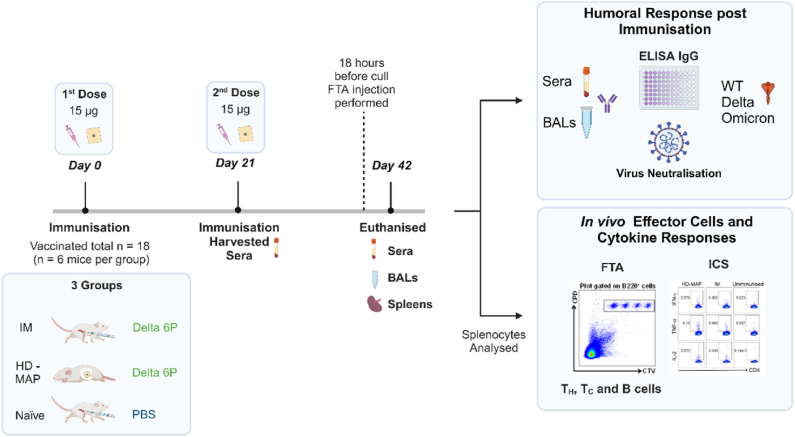
A schematic of the experimental timeline and groups. Mice were immunised via intramuscular (IM), high-density microarray patch (HD-MAP) or PBS only on day 0 and day 21. Serum was harvested on days 21 and 42 for analysis. On day 42, bronchoalveolar lavage (BAL) fluid and spleens were harvested for downstream analysis. Splenocytes were analysed via fluorescent target array (FTA) or intracellular cytokine staining (ICS). Serum and BAL fluid was analysed for IgG titre against an early viral isolate termed WT, a Delta virus isolate and an Omicron virus isolate. Neutralisation was analysed again the cognate Delta virus isolate.

Naïve female BALB/c mice (n = 6 per group) were randomly assorted into experimental group specific cages before proceeding with preparation for immunisation with vaccine formulation. Mice received immunisations (15 μg of plasmid) on day 0 and day 21 using HD-MAP application or intramuscular (IM) injection. Naïve mice were left untreated as controls. A schematic of the experimental procedure is provided in Fig. 1.

### 2.7 HD-MAP coating, application, and immunisation

HD-MAPs (Vaxxas Pty Ltd., Australia) were produced by injection moulding medical-grade synthetic polymer to produce microprojections. Before vaccine coating, HD-MAPs were primed as previously described [12]. A vaccine formulation, containing the BioNet Delta 6P plasmid and excipients (5 % methylcellulose and water) was then applied to the patch. Before vaccine-coated HD-MAP application, the flank of the mice was shaved, and depilatory cream (Nair) used to prime vaccination site. The vaccine-coated HD-MAPs were applied as previously described [12]. IM-injected mice received plasmid diluted in water alone.

### 2.8 Intramuscular immunisation of BALB/c mice

For IM immunisation, a dose-matched vaccine formulation relative to the HD-MAP vaccine formulation was used. 50 μL was injected into the flank of each mouse.

### 2.9 Serum and bronchial lavage fluid collection and preparation

Collection of serum occurred on days 21 and 42 post-vaccination to measure antigen-specific IgG antibody titres. Blood was collected in 1.5 mL tubes via tail tip (day 21) and using cardiac puncture (day 42, after euthanasia with CO_2_). Serum was separated from both tail tip and cardiac bleeds as previously described [12].

Bronchoalveolar lavage (BAL) was performed immediately after cardiac puncture (day 42) by dissection of the animal before injecting 400 μL of saline via the trachea using a 1 mL syringe with a 21G catheter attached to the end. The lungs were washed thrice with the BAL fluid to ensure adequate collection of a representative sample, and the fluid collected in a 1.5 mL tube. The BAL fluid was centrifuged at 1000×*g* for 5 min to remove debris. Supernatant was harvested and stored at – 20 °C until analysis [12].

### 2.10 Bronchial alveolar lavage fluid and serum ELISAs

Serum and BAL fluid sample ELISAs were utilised to determine antigen specific IgG specific titres as previously described [37]. Nunc MaxiSorp 96-well flat-bottom plates (Thermo Fisher Scientific, Waltham, Massachusetts, USA) were coated with SARS-CoV-2 spike HexaPro protein (Wild-type, Beta, or Delta) or Delta RBD and NTD spike protein domains, in 50 μL of 1 × PBS at a concentration of 2 μg/mL overnight at 4 °C. Plates were incubated with blocking buffer (1 × PBS, 0.05 % Tween-20 and 1:20 dilution of KPL Milk diluent/Blocking solution concentrate (Sera Care, Milford, Massachusetts, USA)) for 1 h at room temperature. After blocking buffer was removed, serum was diluted (1:20) and BAL fluid was diluted (1:10) in blocking buffer and a 5-fold serial dilution performed. The sera and BAL fluid ELISAs contained 50 μL/well and were incubated at 37 °C for 1 h. Plates were washed in 0.05 % PBST before adding HRP-conjugated goat anti-mouse antibody (Thermo Fisher Scientific, Waltham, Massachusetts, USA) for 1 h at 37 °C. Absorbance was read and binding curves were analysed for the detection of antigen-specific immunoglobulin G (IgG) binding as described previously [12].

### 2.11 Virus preparations

SARS-CoV-2 virus isolate B.1.617 lineage, hCoV-19/Australia/QLD1893C/2021 (referred to as Delta variant; GISAID Accession ID: EPI_ISL_2433928; collected on May 4, 2021) was provided by the Queensland Health Forensic and Scientific Services, Queensland Department of Health (Queensland, Australia). Virus was passaged using VeroE6-TMPRSS2 cells as previously described [41] and immunoplaque assay utilised to titrate as previously described [42].

### 2.12 Plaque reduction neutralisation tests

The neutralisation activity of mouse sera was assessed via immunoplaque assay as described. Serum was serially diluted in DMEM +2 % FCS before adding virus. After a 30-min incubation at 37 °C, virus-serum complexes were added to 96-well plates containing confluent monolayers of VeroE6 cells. Infection proceeded for 1 h at 37 °C before overlay medium (1 % carboxymethyl cellulose (Sigma Aldrich, Burlington, Massachusetts, USA), 2.5 % FCS (Bovogen, Keilor East, Victoria, Australia) in M199 (Gibco, Los Angeles, California, USA)) was added. Cells were fixed and immunoplaques were visualised as described previously [28,38,42].

### 2.13 Fluorescent target array (FTA)

To measure T cell responses *in vivo*, the FTA protocol that has been previously established was conducted [43]. A combination of the cell tracking dyes, cell proliferation dye eFluor™ 670 (CPD, eBiosciences, San Diego, California, USA) and cell trace violet (CTV, Invitrogen, Waltham, Massachusetts, USA), were used to label autologous and naïve splenocytes before pulsing with SARS-CoV-2 peptides derived from ancestral wild type spike, Delta spike or a mutated (in delta spike relative to wild type spike) pool of peptides (T19R, R158G, L452R, T478K, D614G, P681R, D950N) to make the pulsed cells targets for T cell responses. Spike-specific T helper cells that recognise naïve B cells presenting spike peptides complexed to major histocompatibility (MHC) class II in the FTA array will receive costimulatory signals resulting in activation. This results in the up-regulation of the activation marker, CD69, on cognate peptide presenting B cells. The level of up-regulation of this marker on peptide-pulsed B cells as measured using the geometric mean fluorescent intensity (GMFI) of CD69 in indicative of the magnitude of the Th cell response. CTL mediated recognition of cells in the FTA array that present peptides on MHC class I complexes result in killing of the target cells and the proportion of cells killed relative to mock (nil) is indicative of the magnitude of the CTL response. Spike peptide arrays were obtained from Shanghai RoyoBiotech Co., Ltd. (Shanghai, China) and comprise of peptides that are 15–18 aa in length per peptide with 10–11 aa overlap between adjacent peptides. To prepare the FTA, splenocytes from 10 naïve mice were initially split across 4 tubes and labelled with 22.95, 6.21, 1.67 or 0.595 μM of CTV for 5 min at room temperature, then washed thrice with PBS. Prior to labelling, splenocytes from each tube were resuspended in 1.9 mL of complete medium (Roswell Park Memorial Institute 1640 (RPMI, Gibco, Los Angeles, California, USA) media supplemented with 10 % heat-inactivated foetal calf serum (HI-FCS), 25 mM N-2-hydroxyenthylpiperazine-N-2-ethane sulfonic acid (HEPES, Gibco, Los Angeles, California, USA), 1x 2-mercaptoethanol (Gibco, Los Angeles, California, USA), 1 mM Sodium Pyruvate (Gibco, Los Angeles, California, USA), and 50 U/mL of Penicillin-Streptomycin (Gibco, Los Angeles, California, USA)). Using the same media and supplements, the cells were then washed but with 5 % FCS.

Labelled cells were pulsed with 10 μg/mL with the previously stated mutated peptide pool or individual peptides for 4 h and incubated at 37 °C with 5 % CO_2_. Peptide-pulsed targets were washed thrice as before and labelled with 38.65 μM CPD using the same method for labelling as for CTV. Following this, labelled cells were washed thrice with PBS being used for the final (third) wash and injected intravenously (2 × 10^6^ cells per target cell population in PBS) into immunised mice for the FTA challenge. 18 h after the FTA challenge, red blood cell (RBC)-depleted splenocytes from immunised mice were isolated and analysed using the BD FACSymphony™ A5 SE flow cytometer as described previously [43]. Analysis of data was conducted using FlowJo™ 10.8.1 software. Data were then processed as we have previously described to inform the vaccine-specific T helper and cytotoxic T cell responses of FTA-challenged mice [43].

### 2.14 Intracellular cytokine staining (ICS) analysis

RBC-depleted splenocytes (4 × 10^6^ cells per stimulation in 200 μL of complete medium) from mice at the study end point were stimulated with 2 μg/mL of Delta spike peptide array or DMSO (mock) using a 96-well U-bottom well plate (Thermo Fisher Scientific, Waltham, Massachusetts, USA) at 37 °C with 5 % CO_2_. One hour after the stimulation, brefeldin A (1000× from BioLegend, San Diego, California, USA) was added in a 20 μL bolus of complete medium such that its final concentration in culture was 1× (1000-fold diluted from the original stock). The cultures were then stimulated for 5 more hours prior to viability, cell surface and and intracellular staining being performed using protocol described for McMillan et al. [44]. Fixable viability stain 440 (Becton Dickinson (BD) Biosciences, Franklin Lakes, New Jersey, USA) was used to stain for dead cells and exclude them from analysis. Cell surface staining was performed using phycoerythrin (PE) conjugated CD4 (clone GK1.5, BD Biosciences, Franklin Lakes, New Jersey, USA) and brilliant violent 650 conjugated CD8 (clone 53-6.7, BioLegend, San Diego, California, USA). Intracellular staining was performed using the following antibodies: Alexa Fluor 488 conjugated interleukin (IL)-2 (clone JES6-5H4, BioLegend, San Diego, California, USA), Allophycocyanin/Fire™ 750 conjugated interferon-gamma (IFN-γ) (clone XMG1.2, BioLegend, San Diego, California, USA) and PE-Cy7 conjugated tumour necrosis factor (TNF)-α (clone MP6-XT22, BioLegend, San Diego, California, USA). For discussion purposes, multifunctional or polyfunctional spike-specific CD4^+^ and CD8^+^ T cells are defined as cells producing 2 or 3 cytokines, respectively.

### 2.15 Diagrams, graphs, and statistical analysis

Diagrams for study timeline were designed using BioRender, Canada. GraphPad Prism version 10.1.1 for Windows (GraphPad software, Boston, Massachusetts, USA) was used to construct graphs and P values were calculated using GraphPad Prism or IBM SPSS statistical analysis software as previously described [43].

## 3 Results

### 3.1 HD-MAP delivery of the Delta 6P plasmid induces robust humoral immune responses

**Fig. 2:**
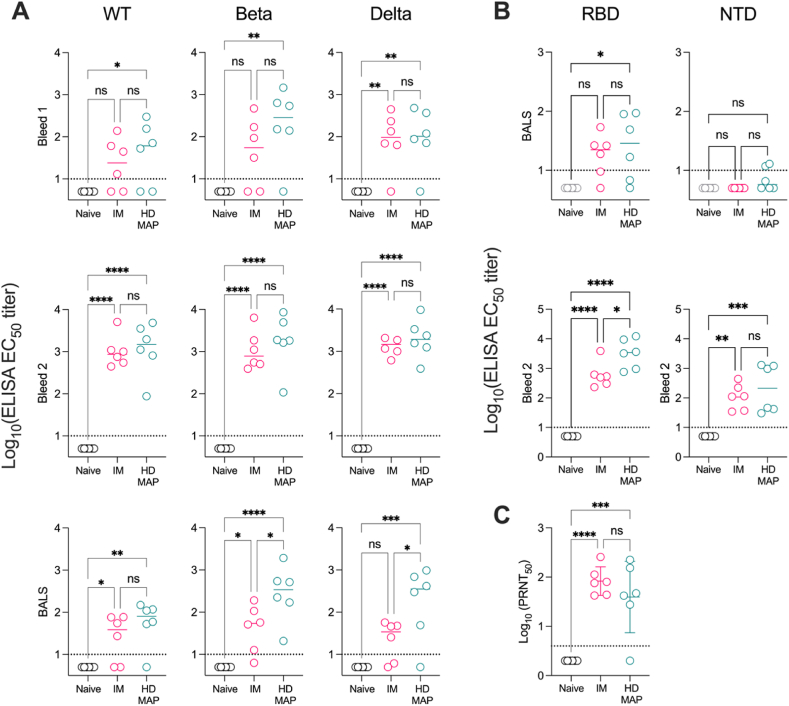
Vaccination with HD-MAP or IM injection induces comparable antibody responses to SARS-CoV-2 spike protein. (A) Analysis of anti-spike IgG titres were performed on bleeds taken on days 21 (bleed 1) and 42 (bleed 2 and bronchoalveolar lavage (BAL) fluid) against the wild type (WT), Beta and Delta spike variants. (B) Analysis of anti-spike IgG titres were performed on day 42 (bleed 2 Serum and BAL fluid) against SARS-CoV-2 N-terminal domain (NTD) and receptor binding domain (RBD) protein domains. Each data point represents one mouse and the mean presented as a line, with any values assessed to be below the limit of detection (LoD) annotated as half the LOD. (C) Virus neutralisation was assessed by plaque reduction neutralisation test (PRNT) against SARS-CoV-2, delta variant isolate hCoV-19/Australia/QLD1893C/2021 (GISAID Accession ID EPI_ISL_2433928). Mouse sera was collected on day 42 (bleed 2) and analysed for virus neutralisation by PRNT. Each data point represents one mouse and the mean represented by a bar for each group. Statistical significance was tested using a one-way ANOVA with a Tukey's multiple comparisons test, post hoc (P values). ^ns^ p > 0.05, ∗ ≤0.05, ∗∗p < 0.01, ∗∗∗p < 0.001.

First, we investigated the immunogenicity of the Delta 6P plasmid in mice, either via HD-MAP application (Fig. 1) or IM injection. Collection of serum occurred on day 21 (bleed 1) and 42 (bleed 2) for assessment of humoral immune responses against the spike protein from three different spike proteins – ancestral HexaPro spike, encoding the sequence of an early SARS-CoV-2 isolate (termed Wildtype, WT), spike protein from the Beta variant, and spike protein from the Delta variant (i.e. matched to the vaccine variant). After the first immunisation, HD-MAP delivery of Delta 6P produced significantly higher serum IgG levels against WT (P ≤ .05) and Beta (P ≤ .01) spike compared to the naïve group. Against the Delta spike, delivery of Delta 6P by either HD-MAP (P ≤ .01) or IM (P ≤ .01) saw a significant increase of IgG titres compared to the naïve mice. After the second dose of Delta 6P, we observed significantly boosted serum IgG responses in the HD-MAP group against WT (P ≤ .0001), Beta (P ≤ .0001), and Delta (P ≤ .0001) proteins relative to the control group (Fig. 2A). Similarly, this trend was reflected in the IM injection group against all spike variants (WT, Beta, and Delta) when compared to the control group (Fig. 2A).

We next investigated the immune response at the lung mucosal surface in BAL fluid collected from the mice on day 42. Here, mice vaccinated via the HD-MAP tended to have higher BAL IgG titres than their IM counterparts (Fig. 2A), though there was no statistically significant difference between the two modalities. However, the HD-MAP group had significantly higher BAL IgG titres relative to the naïve group when tested against all spike proteins, which is in contrast to the IM groups where higher IgG titres than the naïve group were only observed against the Beta spike protein.

To further assess the IgG responses induced by the Delta 6P plasmid, we evaluated serum and BAL fluid collected on day 42 (i.e. after two doses) for IgG responses against the spike protein N-terminal domain (NTD) and receptor-binding domain (RBD) (Fig. 2B). The BAL fluid showed a significant (P ≤ .05) IgG response to the RBD solely in the HD-MAP immunised group as opposed to the IM injected and naïve groups. In contrast, only modest responses were seen to the NTD, with just two out of six mice in the HD-MAP group showing detectable titres and no mice from the naïve or IM groups reacting. When comparing RBD-specific IgG titres within serum, responses were significantly higher compared to the naïve group in both the HD-MAP (P ≤ .0001) and IM (P ≤ .0001) injected groups. Here, we also note the HD-MAP immunised group has significantly higher (P ≤ .05) IgG titres than that of the IM injected group (Fig. 2B). Similarly, when measuring responses against the NTD in the serum, we observed significant IgG responses in both IM (P ≤ .01) and HD-MAP (P ≤ .001) immunised mice comparative to the unimmunised mice.

To assess functional IgG responses, we performed a plaque reduction neutralisation test (PRNT) using serum from bleed 2. Here, both the IM and HD-MAP groups showed significantly higher neutralisation titres relative to the naïve group (Fig. 2C). However, no statistical difference was seen between the two vaccine delivery modalities.

### 3.2 Delta 6P delivery induces comparable cellular responses when delivered via HD-MAP and injection

**Fig. 3:**
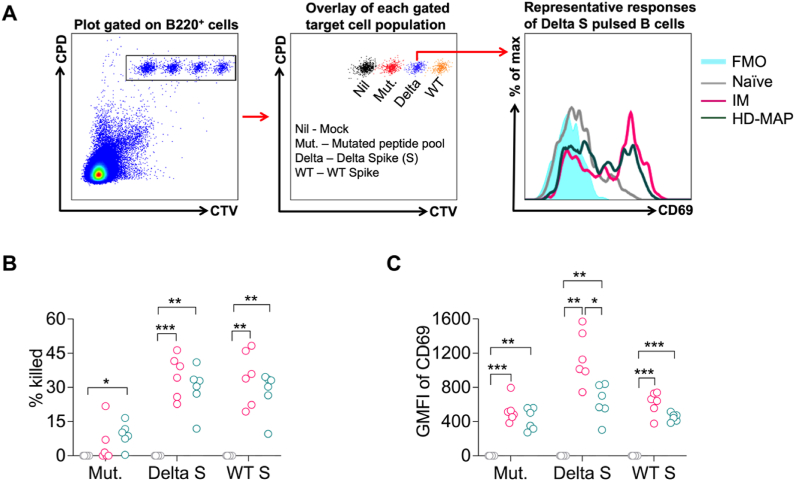
Comparable T helper cell and cytotoxic T lymphocyte responses between IM and HD-MAP Delta 6P vaccinated mice. Mice (n = 6 per group) were vaccinated via IM or HD-MAP with delta 6P DNA as shown in Fig. 1. FTA challenge was performed on day 41 and analysis was conducted on day 42. (A) Representative dot plots show the FTA recovered from a placebo mouse and the peptide pools used to pulse each cell population to make them targets for T cell responses. Nil or unpulsed targets were used as mock controls to measure background responses. Histogram plot shows representative Th cell responses from each group based on the expression of CD69 on naïve B cell targets in the FTA pulsed with Delta Spike peptide array. Mean cytotoxic T lymphocyte (CTL) (B) and Th cell (C) responses specifically elicited against the peptide-pulsed targets used for the FTA challenge. GMFI, geometric mean fluorescence intensity. Welch's ANOVA with Games-Howell, post-hoc test comparisons yielded statistical significance (P values). ^ns^ p > 0.05, ∗ ≤0.05, ∗∗p < 0.01, ∗∗∗p < 0.001.

Next, we sought to evaluate the T cell immunity elicited following vaccination with the Delta 6P plasmid. Firstly, we utilised the FTA protocol to obtain a real-time snapshot of the magnitude of the T helper cell and CTL responses *in vivo* (Fig. 3A). Data showed that Delta 6P vaccination via the IM or HD-MAP delivery routes elicited comparable spike-specific CTL response to each target cell population analysed with responses to Delta and wild type spike being the most robust (Fig. 3B). Th cell response followed a similar trend although Delta spike-specific response was the higher in the IM group compared to the HD-MAP (Fig. 3C).

**Fig. 4:**
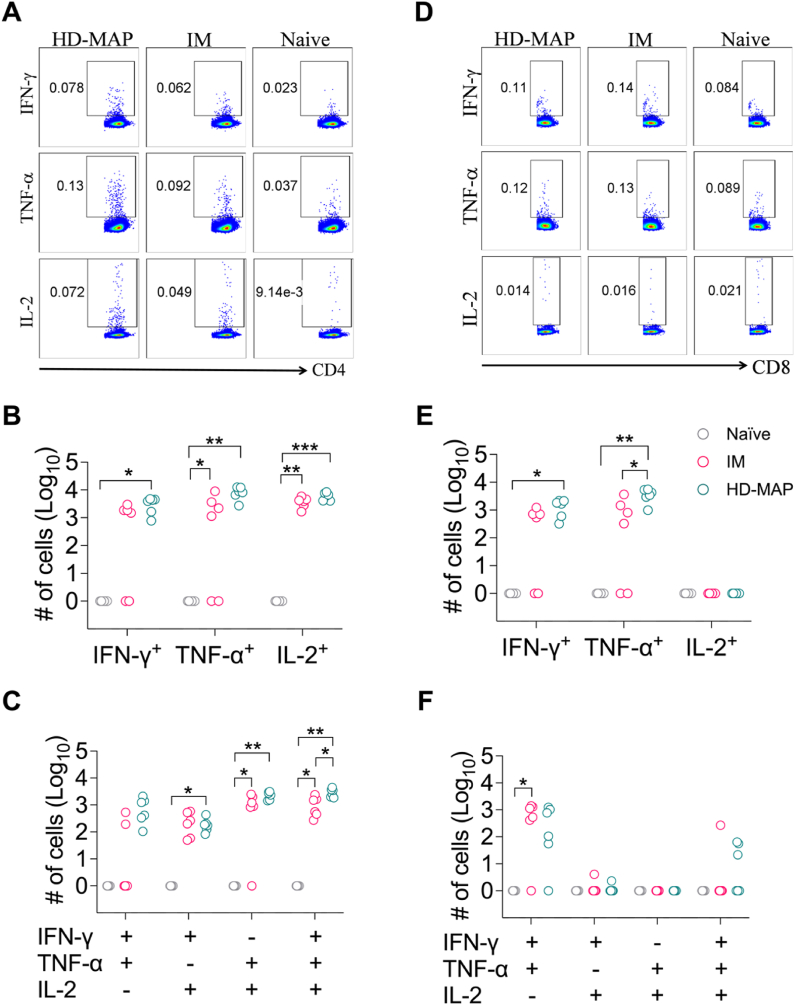
CD4^+^ and CD8^+^ T cell-mediated Spike-specific cytokine responses elicited following vaccination. Mice were vaccinated or treated as shown in Fig. 1 and at the study end point (day 42) T cell responses in the spleen were evaluated following stimulation with Delta S. Dot plots show the gating strategy for determining the intracellular expression of IFN-γ, TNF-α, and IL-2 in gated CD4^+^ (A) and CD8^+^ (D) T cells. (B, C) CD4^+^ T cell responses and (E, F) CD8^+^ T cell responses were measured. Each data point represents an individual mouse response, and the p values were tabulated as described in the Methods using SPSS. ^ns^ p > 0.05, ∗ ≤0.05, ∗∗p < 0.01, ∗∗∗p < 0.001.

Next, we utilised the ICS assay to investigate the T cell-mediated spike-specific cytokine responses (Fig. 4A, D). We focused the analysis on IFN-γ, TNF-α and IL-2 given that the magnitude of T cells that produce these cytokines correlates with protection against intracellular pathogens and viruses. Following analysis of splenocytes stimulated with Delta spike peptides, we observed robust spike-specific CD4^+^ T cell responses producing IFN-γ, TNF-α or IL-2 following HD-MAP and IM vaccinations (Fig. 4B). The spike-specific CD8^+^ T cell response was more subtle compared to the CD4^+^ T cell response with no IL-2 producers in both vaccination cohorts (Fig. 4E).

We then examined the multifunctional or polyfunctional spike-specific CD4^+^ and CD8^+^ T cells. The polyfunctional CD4^+^ T cell response in this instance was mostly comparable between IM and HD-MAP groups although we observed a statistically significant enhancement in the number of triple cytokine producers in the HD-MAP group compared to IM (Fig. 4C). With respect to CD8^+^ T cell polyfunctionality, only IFN-γ and TNF-α double producer subset was observed to be higher in the vaccination groups relative to placebo. However, there was no difference in the magnitude of this subset between IM and HD-MAP groups (Fig. 4F).

Overall, the analysis of the T cell response *in vivo* and *in vitro* suggest that Delta 6P plasmid vaccination delivered via the HD-MAP or IM elicited robust, yet mostly comparable spike-specific responses in both the Th/CD4+ and CTL/CD8+ T cell compartments.

## 4 Discussion and conclusion

SARS-CoV-2 continues to be a major health burden worldwide. Here, we report an alternative vaccination strategy using a DNA vaccine targeting the spike protein, delivered to the skin via a microarray patch, the HD-MAP. This work utilised a vaccine encoding the spike protein from the Delta variant of SARS-CoV-2. While the Delta variant is no longer globally circulating, this vaccine candidate was chosen as a model antigen, designed to demonstrate a proof-of-concept for the DNA vaccine approach as well as HD-MAP delivery. To provide more robust protection against recent variants, it is likely that future variations would need to be updated to match the new SARS-CoV-2 spike protein sequences. DNA vaccines allow for rapid updating to match circulating variants, however, owing to their ease of manufacturing and the fact that only sequence information is required, which is available in abundance in the case of SARS-CoV-2.

Delivery of vaccines via the HD-MAP has the potential to offer an alternative vaccine strategy that is safe and easy to administer, even allowing the possibility for self-administration. This work showed that delivery of this DNA vaccine via the HD-MAP resulted in potent induction of anti-spike antibodies in mice, in similar levels to those induced by IM injection (Fig. 2). The antibody responses were measured against subdomains of the spike protein, with very little reactivity against the NTD relative to the RBD. While antibodies targeting the S2 domain were not directly measured here, it is known that the RBD domain is immunogenic and the target for potent neutralising antibodies [45]. IgG measurements against the whole spike protein revealed higher response compared to the RBD alone. Therefore, it is logical to conclude antibodies against domains other than the RBD and NTD (i.e. the S2 domain) were induced by vaccination with this DNA vaccine candidate. The spread of IgG responses in the HD-MAP group could be considered to be slightly broader than the IM group. This could reflect the delivery method, with perhaps less consistent delivery via the HD-MAP than controlled injection via injection. To assess this, scanning electron microscopy could be used to evaluate delivery of the vaccine to the skin, and potential changes in the vaccine formulation could be applied to achieve consistent delivery. To assess functionality of the induced antibody response, we performed virus neutralisation assays. We observed neutralisation in both IM and HD-MAP groups (Fig. 2C), though one animal in the HD-MAP group did not mount a neutralising response. While antibodies directed towards the S1 subunit of the spike protein, containing the RBD and NTD domains, work by inhibiting virus attachment to host cells thereby preventing infection, these domains are often mutated in emerging variants. Thus, antibodies directed against the S2 domain, which is much more conserved, have the potential to have broader reactivity [46]. S2-specific monoclonal antibodies have been shown to provide protective efficacy through Fc-mediated effector functions such as antibody-dependent cellular cytotoxicity and antibody-dependent cellular phagocytosis [47]. Therefore, it is possible that *in vivo* challenge studies are needed to fully evaluate the impact of non-neutralising antibodies induced by the Delta 6P vaccination.

As SARS-CoV-2 infects via the respiratory tract, we measured the IgG response in BAL fluid of vaccinated mice. While we observed responses in both IM and HD-MAP groups, it appeared to trend higher in the latter. This is in line with previous HD-MAP vaccination studies by our group, where delivery of protein vaccines induced higher BAL fluid IgG responses compared to injection comparators [37]. While the exact mechanism of this is unknown, it is possible that the site of delivery to the skin via the HD-MAP is inducing more potent mucosal immune responses that result in IgG being present in the lung. Prior studies demonstrated that cutaneous vaccination resulted in primed dendritic cells migrating to mucosal sites to present antigen to resident lymphocytes, resulting in mucosal immunity at a site different to that of vaccination [48].

Human studies have revealed that in contrast to neutralisation antibody epitopes of the spike, T cell epitopes of the spike are more abundant and facilitate more effective cross-recognition across variants [[49], [50], [51]]. It is widely recognised that the helper activity of CD4^+^ T cells is crucially required for eliciting a long-lived antibody response and licensing/mobilising CD8^+^ T cell response that function to effectively control virus infection. Using two independent assays, namely FTA and ICS, we analysed multiple facets of the T cell response *in vivo* and *in vitro* to elucidate the most effective vaccination regimen. We have performed this dual analysis in the past to effectively delineate a vaccination regimen for testing in Phase I clinical trials against SARS-CoV-2 [51,52]. The importance of this type of dual analysis is that the use of a singular assay analysing a certain functionality of T cells may result in the researcher to erroneously make conclusions regarding the most effective vaccination regimen. For instance, if we only used the ICS analysis in this study as is the case with numerous studies in the literature evaluating T cell immunity, we could have concluded that HD-MAP vaccination is more robust than IM in eliciting T cell responses. The use of two assays, each analysing different functions to T cells allowed us to observe that this is not the case.

T cell analyses in the current study showed that Delta 6P plasmid vaccination elicited robust T cell response in both IM and HD-MAP groups (Fig. 3). The responses encompassed cytotoxicity, productive helper activity to activate naïve B cells with the potential to produce antibodies and polyfunctionality, all of which are desirable for the control of virus infection including SARS-CoV-2 [53].

While this study only investigated delivery to mouse skin, human skin has a thicker epidermis and dermis, which could have implications for vaccination via the HD-MAP [54]. There have been multiple human clinical trials performed with the HD-MAP in humans, where the same increases in immunogenicity relative to injections are observed. For example, delivery of an influenza virus vaccine showed increases in immunogenicity compared to IM injection, and the same immune responses when a 6-fold lower dose was delivered via the HD-MAP [24]. Similar trends were seen with a measles and rubella vaccine delivered via the HD-MAP [55]. These studies did not use DNA vaccines, so further work with this vaccine modality would be required in humans to determine if the same immunogenicity trends are seen with thicker, human skin. In these studies, it could be beneficial to include electroporation after the IM injection, which can result in more efficient DNA vaccination. It is hoped that the use of the HD-MAP will overcome the need for difficult-to-use electroporation equipment, however this would need to be tested in a head-to-head study.

Overall, this study highlights a viable alternative vaccination study against SARS-CoV-2 that is capable of producing both cellular and humoral immunity. Delivery via the HD-MAP provides an alternative delivery mechanism allowing ease of administration and distribution while inducing mucosal immune responses, which have potential to be beneficial in the context of SARS-CoV-2 infection. Further challenge studies could provide further evidence for the utility of this vaccine candidate.

## CRediT authorship contribution statement

**Christopher L.D. McMillan:** Writing – review & editing, Writing – original draft, Visualization, Methodology, Investigation, Conceptualization. **Andrea V. Corner:** Writing – review & editing, Writing – original draft, Visualization, Methodology, Investigation, Conceptualization. **Danushka K. Wijesundara:** Writing – original draft, Visualization, Methodology, Investigation, Conceptualization. **Jovin J.Y. Choo:** Writing – original draft, Investigation, Conceptualization. **Daraporn Pittayakhajonwut:** Writing – review & editing, Methodology. **Indrajeet Poredi:** Writing – review & editing, Methodology. **Rhys H. Parry:** Writing – review & editing, Methodology. **Guneet K. Bindra:** Methodology. **Kimberley L. Bruce:** Methodology. **Alexander A. Khromykh:** Supervision. **Germain J.P. Fernando:** Writing – review & editing, Validation, Conceptualization. **Laurent Dapremont:** Writing – review & editing, Supervision, Conceptualization. **Paul R. Young:** Supervision. **David A. Muller:** Writing – review & editing, Supervision, Conceptualization.

## Ethics statement

All animal procedures and protocols were approved and conducted in accordance with the University of Queensland Animal Ethics Committees (AEC), AEC approval number: 2021/AE000340.

## Data availability

Data will be made available upon request.

## Funding information

This work was supported by the Advance Queensland Industry Research Fellowship 2020001511 (DAM), sponsored by Vaxxas and Technovalia.

## Declaration of competing interest

The authors declare the following financial interests/personal relationships which may be considered as potential competing interests:David A. Muller, Paul R. Young, Danushka K. Wijesundara, Germain J.P. Fernando, Christopher L.D. McMillan reports a relationship with Vaxxas Pty Ltd that includes: consulting or advisory, employment, and funding grants. Laurent Dapremont reports a relationship with Technovalia that includes: employment. Indrajeet Poredi, Daraporn Pittayakhajonwut reports a relationship with BioNet-Asia Co Ltd that includes: employment. If there are other authors, they declare that they have no known competing financial interests or personal relationships that could have appeared to influence the work reported in this paper.
